# Infarctus du myocarde révélateur d'une thrombocytémie essentielle chez un sujet jeune noir africain: à propos d'une observation

**DOI:** 10.11604/pamj.2014.18.347.5201

**Published:** 2014-08-29

**Authors:** Nobila Valentin Yaméogo, Larissa Justine Kagambèga, Aimé Arsène Yaméogo, Koudougou Jonas Kologo, Georges Rosario Christian Millogo, Boubacar Jean Yves Toguyéni, André Koudnoaga Samadoulougou, Patrice Zabsonré

**Affiliations:** 1 Service de Cardiologie, CHU-Yalagado Ouédraogo, Ouagadougou, Burkina Faso; 2 Service de Cardiologie, CHU-Sanou Souro, Bobo Dioulasso, Burkina Faso

**Keywords:** Thrombocythémie essentielle, infarctus du myocarde, sujet jeune, Ouagadougou, essential thrombocythemia, myocardial infarction, young man, Ouagadougou

## Abstract

La thrombocytémie essentielle est un syndrome myéloprolifératif qui se complique rarement d'infarctus du myocarde. Nous rapportons l'observation d'un patient de 23 ans, sans facteurs de risque cardio-vasculaire connus, aux antécédents de thrombose veineuse cérébrale à l’âge de 20 ans, admis dans le service de cardiologie pour un syndrome douloureux thoracique. L'examen physique était pauvre. L'ECG, la troponinémie et la coronarographie ont conclu à un infarctus du myocarde par obstruction distale de l'IVA. La numération formule sanguine objectivait une importante thrombocytose isolée à 1.197.000/mm3. La recherche de la mutation V617F de JAK2 était positive. Il n'y avait pas de thrombophilie. L’évolution était favorable sous héparine de bas poids moléculaire, antiagrégant plaquettaire, hydroxyurée et hydratation alcaline abondante.

## Introduction

La thrombocytémie essentielle est un syndrome myéloprolifératif chronique caractérisé par une élévation durable du chiffre des plaquettes supérieur à 450 000/mm3 [1]. Le diagnostic est fait après exclusion des thrombocytoses réactionnelles (syndrome inflammatoire, carence martiale ou asplénie) et des autres syndromes myéloprolifératifs [2]. Le marqueur moléculaire principal des syndromes myéloprolifératifs en l'absence du chromosome Philadelphie est la mutation V617F du gène JAK2. Cette mutation est présente dans la plupart des polyglobulies primitives et dans la moitié des thrombocytémies essentielles [2, 3]. L'infarctus du myocarde est une complication rare de la thrombocyhémie essentielle [4]. Nous présentons le cas d'un sujet jeune de sexe masculin dont la circonstance de découverte était un infarctus du myocarde.

## Patient et observation

Monsieur HS 23 ans, sportif de haut niveau est admis dans le service de cardiologie pour des douleurs thoraciques d'allure angineuse survenues au repos et évoluant depuis 12 heures. L'anamnèse revèle des épisodes douloureux thoraciques suggestives d'angor d'effort depuis deux (02) mois environs et une thrombophlébite cérébrale à l’âge de 20 ans. Il n'y a pas d'intoxication alcoolo-tabagique ni de dopage. Il n'est pas rapporté un antécédent de syndrome hémorragique ni de fièvre.

**Figure 1:**
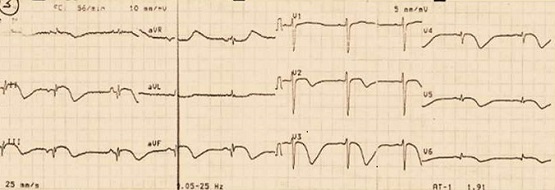
ECG de surface à 12 dérivations; rythme sinusal régulier avec une ischémie-lésion sous épicardique et nécrose en inférieur, et une ischémie sous épicardique en antérieur étendu

A l'admission, la tension artérielle était de 100/60 mmHg, la fréquence cardiaque à 62 battements/min, le poids à 64 kg et la taille 180 cm. Il n'y avait pas de fièvre. L'examen physique en particulier cardio-vasculaire était pauvre. Il n'existait ni hépato- splénomégalie ni adénopathie périphérique. L'ECG réalisé en urgence (Figure 1) montrait une ischémie-lésion sous épicardique en inférieur, une nécrose dans le même territoire et une ischémie sous épicardique en antérieur étendu. La radiographie pulmonaire était normale de même que l’échoDoppler cardiaque. Le troponine I était élévée à 100 fois la normale.

**Figure 2:**
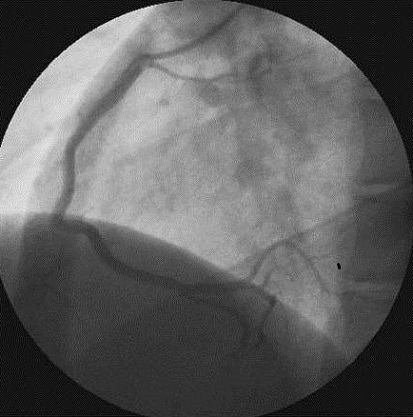
Coronarographie droite en OAG. La coronaire droite (dominante) est angiographiquement saine

**Figure 3:**
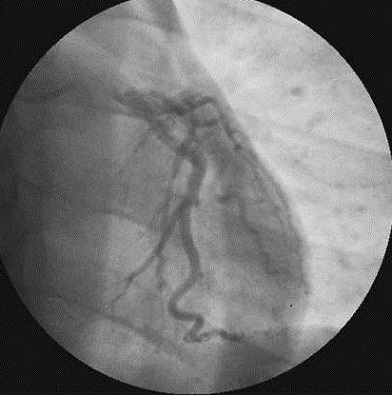
Coronarographie gauche incidence face craniale. Obstruction distale de l'IVA. (Flèche)

La numération formule sanguine objectivait une importante thrombocytose isolée à 1.197.000/mm3. Les globules blancs étaient à 9190/mm3 avec 4328 neutrophiles/ mm3. Le taux d'hémoglobine était normal de même que les globules rouges (5.190.000/ mm3). Les sérologies VIH, hépatitique et syphilitique étaient négatives. Le bilan inflammatoire (CRP et VS) était normal. La recherche de thrombophilie (dosage de la protéine C, de la protéine S, de l'antithrombine, recherche de mutation du facteur V Leiden) était négative. Le bilan martial, l’électrophorèse des protides sériques et la recherche d'anticorps antiphospholipides n'avaient pas objectivé d'anomalies. La recherche d'anticorps antinucléaires et d'anticorps anti DNA natif était négative. Le myélogramme concluait à un aspect très dilué de la moelle montrant de très rares mégacaryocytes, de rares précurseurs médullaires (granuleux et érythroblastiques) et de nombreux neutrophiles évocateurs d'un syndrome myéloprolifératif. La biopsie ostéomédullaire retrouvait un tissu hématologique de richesse 3 à 4 où toutes les lignées y sont representées sans anomalie de maturation. On y notait essentiellement des mégacaryocytes nombreux et géants, aux noyaux hyper-multilobés à disposition paratrabéculaire et péri-sinusale. L’étude de la masse sanguine se révélait normale, et la recherche de la mutation V617F de JAK2 était positive, confirmant le diagnostic de thrombocytémie essentielle. La coronarographie (Figure 2, Figure 3) mettait en évidence une occlusion distale de l'interventriculaire antérieure qui a été respectée. Sous traitement à base d'héparine de bas poids moléculaire, d'antiagrégant plaquettaire, myéloréducteur (hydroxyurée), hypo-uricémiant associés à une hydratation alcaline abondante l’évolution a été favorable et le patient est mis en éxéat après une hospitalisation de 10 jours. Il est actuellement suivi au CHU-YO.

## Discussion

La thrombocytémie essentielle est un syndrome myéloprolifératif qui ne modifie pas de façon significative l'espérance de vie des sujets atteints. La morbidité et la mortalité liées aux complications vasculaires, hémorragiques ou thrombotiques, sont néanmoins notables dans cette population [5]. En effet, 20 à 50% des patients présentent une complication thromboembolique au moment du diagnostic [3, 6, 7]. Ces complications thromboemboliques sont moins fréquentes chez les sujets de 60 ans et augmentent au-delà de 70 ans. Les complications hémorragiques sont observées dans 16 à 20% des patients environs [7]. Notre patient a présenté une thrombose veineuse cérébrale à l’âge de 20 ans mais le diagnostic de thrombocytémie essentielle n'avait pas été établi. L'infarctus du myocarde est une complication rare de la thrombocytémie essentielle. Rossi en Italie trouvait une prévalence de 9,4% [4]. La particularité de notre observation est la survenue précoce de l'infarctus myocardique parce qu'en général il survient au-delà de 40 ans dans cette pathologie [4]. Les accidents vasculaires cérébraux sont plus fréquents dans la thrombocythémie essentielle. Ils touchent 22% des patients [8]. La thrombophlébite cérébrale est rencontrée dans 9,7% des cas. Plusieurs théories ont été postulées pour expliquer le mécanisme de la thrombose coronaire liée à la thrombocytose. Il s'agit d'une anomalie de l′activation du système fibrinolytique, d'un renforcement de l'activité procoagulante des plaquettes et une augmentation de la viscosité sanguine [9, 10]. Des études antérieures ont montré que c'est la taille des plaquettes plutôt que leur nombre absolu qui était corrélée avec les complications thrombotiques. L'inhibition de l'agrégation plaquettaire et de la production des plaquettes joue un rôle important dans le traitement de la thrombocytémie essentielle compliquée de thrombose coronaire. Certains auteurs utilisent à cet effet les inhibiteurs des récepteurs des glycoprotéines IIb/IIIa (abciximab) [10]. Cependant les anti-plaquettaires chez ces patients sont incriminés dans la survenue des complications hémorragiques plus qu'ils ne préviennent la thrombose. Néanmoins, une faible dose d'aspirine peut réduire le risque thrombose coronaire sans augmenter les complications hémorragiques [4]. Chez notre patient, une faible dose d'aspirine a été introduite dans le traitement (100mg). L'hydroxyurée représente le traitement cytoréducteur standard. L'efficacité de ce médicament dans la réduction des complications thrombo-emboliques a été démontrée [6, 9].

Du point de vue pronostic l'infarctus du myocarde en rapport avec cette pathologie n'est pas moins grave que dans la maladie athéromateuse. La prévention du risque d'accidents thrombotiques ou emboliques doit éliminer tout facteur de risque vasculaire en l'occurrence le tabagisme; l'hypertension artérielle; le diabète; la surcharge pondérale et les dyslipidémies secondaires. Notre patient ne présentait aucun risque vasculaire associé. L’évolution a été simple.

## Conclusion

La thrombocytémie essentielle est une cause rare d'infarctus du myocarde. La recherche d'un syndrome myéloprolifératif doit être entreprise dans les cas d'infarctus survenant dans un contexte particulier comme c'est le cas chez notre patient (sujet jeune avec antécédent de thrombophlébite cérébrale). Le traitement repose sur l'aspirine à faible dose et l'hydroxyurée. La surveillance doit être rigoureuse afin de prévenir les complications thrombo-emboliques et hémorragiques.
